# Understanding Fast and Slow Signal Changes in a Competitive Particle-Based Continuous Biosensor

**DOI:** 10.1021/acs.analchem.5c01457

**Published:** 2025-06-10

**Authors:** Sebastian Cajigas, Arthur M. de Jong, Junhong Yan, Menno W. J. Prins

**Affiliations:** † Department of Biomedical Engineering, 3169 Eindhoven University of Technology, 5612 AZ, Eindhoven, The Netherlands; ‡ Department of Applied Physics and Science Education, Eindhoven University of Technology, 5612 AZ, Eindhoven, The Netherlands; § Institute for Complex Molecular Systems (ICMS), Eindhoven University of Technology, 5612 AZ, Eindhoven, The Netherlands; ∥ Helia Biomonitoring, 5612 AR, Eindhoven, The Netherlands

## Abstract

The development of continuous biosensing technologies requires studies on time-dependent changes in sensor properties because such changes can impact the analytical performance of the sensor. In previous work, we studied long-term changes of a continuous cortisol sensor based on particle motion, which highlighted the roles of molecular loss processes in the biosensor. In this work, we study a glycoalkaloid sensor and observe two characteristic behaviors, namely fast and slow signal changes. Experiments were performed with single-sided aging, motion pattern analysis, and different blocking conditions. The leading hypotheses from this paper are that (i) fast signal changes predominantly result from multivalent interactions between the particle and the sensing surface, and (ii) slow signal changes arise from the gradual dissociation of analogue molecules from the sensing surface. The results give pointers for enabling long-term continuous sensing using particle-based biosensors.

## Graphical abstract

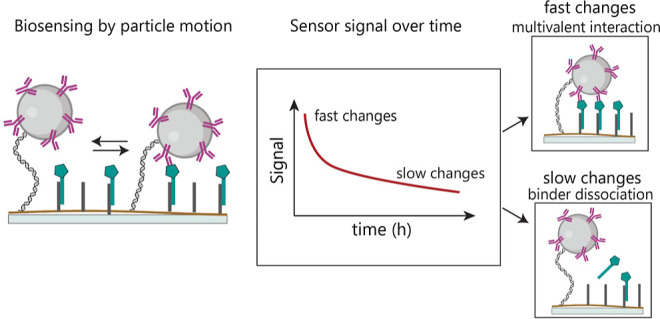

## Introduction

Continuous biosensing technologies are being developed to enable the serial collection of biochemical measurement data over long durations.^1,2^ Such continuous sensors will be suited for monitoring time-dependent processes across diverse applications, including fundamental biological research,^3–5^ patient care,^6,7^ organ-on-a-chip studies,^8,9^ bioreactors,^10,11^ environmental monitoring,^12^ and industrial process control.^13,14^ To achieve continuous biosensing of analyte molecules at micromolar concentrations and below, sensing technologies are being investigated based on nanoscale structures that switch between distinct states in response to reversible affinity-based binding of analyte molecules. The switching behavior can be detected using electrochemical,^15,16^ fluorescence^17,18^ or particle-based^19–21^ approaches. Regardless of the readout modality, the time-dependencies of sensor signals need to be characterized and understood in detail, as these critically impact the analytical performance that can be achieved with the continuous sensing principles.^22–25^

Biosensing by particle motion (BPM) is a particle-based continuous sensing methodology with single-molecule resolution.^19,21^ The technique relies on reversible, affinity-based interactions between biofunctionalized particles and a biofunctionalized sensing surface. Hundreds to thousands of particles are continuously monitored using video microscopy, detecting transitions between bound and unbound states, and reflecting binding and unbinding events between affinity binders on the particles and the sensing surface.^19^ The presence of analyte molecules modulates the frequency of state transitions, which is then a measure of the analyte concentration in solution.^26^

BPM is, in principle, suited for measurements over long time spans because the sensing methodology is reversible and does not consume any sensing reagents. However, BPM experiments over periods of hours and days have revealed gradual changes in the sensor properties.^13,26^ In previous work on a cortisol BPM sensor, we introduced methodologies to investigate the molecular origins of such long-term changes, using aging of individual components (particles and sensing surface) and motion pattern analysis.^20^ By aging individual signal-generating components in the cortisol sensor, we identified that the primary causes of long-term changes were the dissociation of antibodies from the particles, leading to an increase in nonspecific interactions over time, and the dissociation of analogue molecules on the sensing surface, resulting in a reduction in the number of switching events.^25^

In this work, we investigate gradual signal changes in a competitive glycoalkaloid BPM sensor. Glycoalkaloids (GAs) are small-molecule compounds that occur naturally in potatoes and need to be monitored during food production processes. Experiments have shown that gradual changes in the GA sensor have two characteristic behaviors, namely, fast and slow signal changes. To elucidate the mechanisms underlying the signal changes, we studied the aging of individual BPM components, analyzed motion patterns, and performed blocking experiments of particles and surfaces. The results suggest that the fast signal changes dominantly stem from multivalent interactions between the particle and the sensing surface, and slow signal changes arise from the gradual dissociation of analogue molecules from the sensing surface. The gained insights will guide the development of affinity-based continuous sensors suited for extended operational lifetimes.

## Materials and Methods

### Experimental Protocols

Protocols are given in the Supporting Information Section S1 for surface and particle biofunctionalization in t-BPM experiments, t-BPM sensor assembly, single-sided aging experiments in f-BPM, and for particle imaging and data analysis in t-BPM and f-BPM experiments.

### t-BPM GA Sensing in Potato Fruit Juice

Raw potato fruit juice with 1406 mg/L total GA concentration (determined by HPLC) was centrifugated at 15,000 rpm for 10 min for the sedimentation of solid materials. After centrifugation, 20 μL of supernatant was diluted 200-fold in PBS containing 0.5 M NaCl, achieving a final total GA concentration of 8.16 μM. Subsequently, the sample was subjected to 2-fold serial dilutions in PBS containing 0.5 M NaCl, resulting in solutions with GA concentrations ranging from 0.24 to 8.16 μM. The GA samples were transported into the cartridge channel at a flow rate of 100 μL/min for 2 min. Subsequently, the particle motion was recorded over 10 consecutive 1 min intervals in the absence of flow.

### Long-Term Signal Changes in GA t-BPM Sensor under Static Conditions

In long-term experiments, the signal of the competitive GA t-BPM sensor was continuously recorded for about 20 h. Measurements were conducted in 0.5 M NaCl in PBS, without analyte in solution and without fluid exchange. The data acquisition involved 10 measurement blocks of 1 min, evenly distributed over the entire measurement period.

### Particle Aging Studied with f-BPM

To study particle aging on f-BPM, *anti*-solanidine antibody-functionalized particles were prepared on different days and suspended in 0.5 M NaCl in PBS at varying concentrations (0.008 and 0.07 mg/mL). The particle suspensions were incubated at RT while rotating for periods ranging from 4 h up to 92 h. After 92 h, both aged and freshly prepared particle suspensions were diluted to a concentration of 0.008 mg/mL and added to freshly prepared sensing surfaces. The effect of particle aging on binding capacity was evaluated by measuring the bound fraction using both direct and competition assay readouts on freshly prepared sensing surfaces.

### Aging of Sensing Surfaces Studied with f-BPM

DBCO-ssDNA capture oligo functionalized flow cells were initially washed with PBS containing 0.5 M NaCl to remove any unreacted capture molecules. Then, a solution with 10 nM ssDNA-solanidine analogue in PBS containing 0.5 M NaCl was added to the flow cells and incubated for 20 min. After incubation, the analogue-functionalized surfaces were either maintained as prepared or subjected to fluid exchange with 0.5 M NaCl in PBS for aging. The aging duration of the sensing surfaces ranged from 4 h up to 92 h. After the aging process, freshly prepared particles at a concentration of 0.008 mg/mL were manually flown into both aged and freshly prepared sensing surfaces and incubated for 30 min to allow sedimentation. The effect of surface aging was evaluated using both direct and competition assay readouts. For the direct assay, freshly prepared particles were suspended in PBS containing 0.5 M NaCl at a particle concentration of 0.008 mg/mL. This solution was then pipetted onto the aged sensing surfaces to facilitate specific interactions between particles and the sensing surfaces. For the competition assay, freshly prepared particles were mixed with a high analyte concentration (16 μM) to block the antibodies on particles, thereby preventing binding interactions. This mixture was subsequently pipetted onto the aged sensing surfaces. In both assays, particles were incubated for about 30 min to allow sedimentation before measuring the bound fraction values.

### Long-Term Signal Changes Studied in t-BPM with Partially Blocked Surfaces

Sensing surfaces of COC cartridges containing tethered particles were partially blocked using different steps. In a first experiment, the surface was simultaneously activated and blocked by flushing 100 μL of a mixed solution containing 10 nM of ssDNA-solanidine analogue and 10 nM of ssDNA oligo blocker (5′-TGG TCT TAC CCC TGC CGC AC 3′). This mixture was incubated for approximately 5 min, followed by flushing with 200 μL of PBS containing 0.5 M NaCl to remove any unbound analogue and oligo blocker molecules. In a second experiment, the surface was first activated by flowing the analogue solution (10 nM), followed by the addition of a high concentration of analyte. After this, unbound analogue and analyte molecules were washed away using PBS with 0.5 M NaCl. In a third experiment, the surface was simultaneously activated and blocked by flowing a mixture of glycoalkaloid-ssDNA analogue and oligo blocker. This was followed by the addition of a high concentration of analyte, after which the cartridge was washed with PBS containing 0.5 M NaCl. Following the sensor preparations, the t-BPM sensor signals were continuously recorded over about 20 h.

## Results

### Particle-Based Continuous Biosensor

Figure 1 illustrates the tethered sensor based on particle motion (t-BPM) studied in this research. Streptavidin-coated particles (1 μm in diameter, Dynabeads MyOne) were functionalized with biotinylated *anti*-solanidine antibodies and partially blocked with biotinylated polyT molecules.

**Figure 1:**
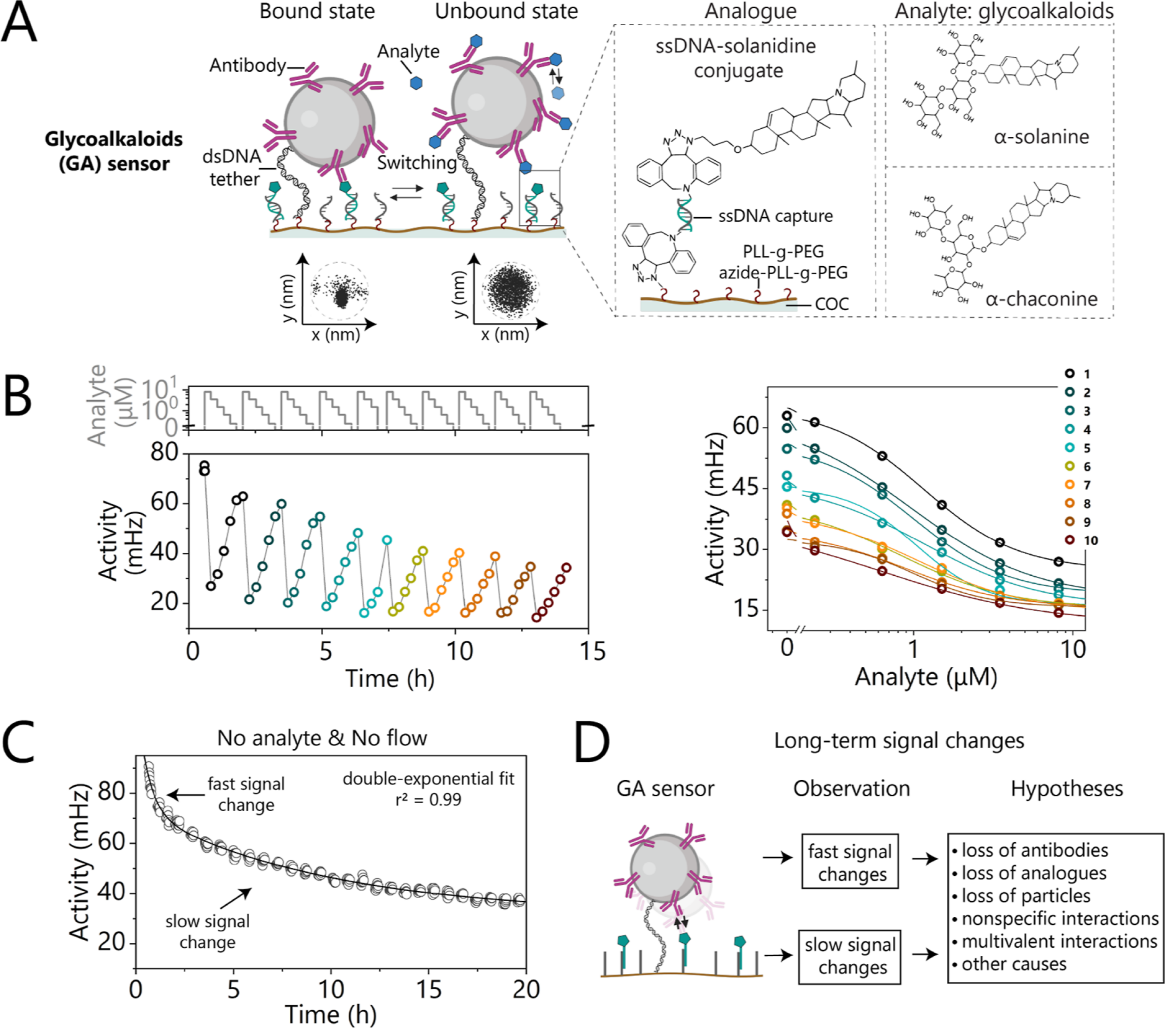
Schematic of a competitive tethered biosensor by particle motion (t-BPM) for continuously monitoring glycoalkaloids (GAs) in potato fruit juice (PFJ). (A) To assemble the GA sensor, the substrate is coated with a low-fouling polymer bottle-brush layer containing PLL-*g*-PEG and azide-functionalized PLL-*g*-PEG to enable the covalent immobilization of DBCO-double strand DNA tether molecules (black) and DBCO-single strand DNA capture oligo (gray). Streptavidin-coated particles are functionalized with a biotinylated *anti*-solanidine antibody (purple) and then attached to the sensing surface via a dsDNA tether molecule through biotin–streptavidin coupling. The analogue molecules, ssDNA conjugated with solanidine (green), are immobilized on the sensing surface through DNA hybridization. The antibody–analogue interaction is reversible, resulting in particles switching between bound and unbound states. The switching events are determined by monitoring the motion of the particles using bright field video microscopy with a dedicated particle-tracking algorithm. (B) The left panel illustrates an example of continuous monitoring of GA in PFJ samples over 15 h. The top panel presents the applied analyte concentration–time profile, with concentrations ranging from 0 to 8.16 μM. The bottom panel displays the switching activity as a function of time, where each point corresponds to 10 consecutive 1 min measurements. The right panel displays the ten dose–response curves derived from the data in the left panel. The data shows that the GA sensor exhibits gradual signal changes over 15 h. (C) Switching activity of the competitive GA t-BPM sensor, without analyte in solution and without any fluid exchange during 20 h. Each activity data point represents a 1 min measurement. The data was fitted with a double-exponential decay equation: *y* = *A*_0_ + *A*_1_·exp­(−*k*_1_·*x*) + *A*_2_·exp­(−*k*_2_·*x*). A fast and a slow decay can be observed in the graph, with loss rates of *k*_1_ = 1.35 ± 0.05 h^–1^ and *k*_2_ = 0.10 ± 0.01 h^–1^, respectively. (D) Potential mechanisms for fast and slow signal changes in a BPM sensor.

The sensing surface was composed of a cyclic olefin copolymer (COC) substrate coated with a low-fouling polymer consisting of poly­(l-lysine)-grafted-poly­(ethylene glycol) (PLL-*g*-PEG) and PLL-*g*-PEG-azide. The azide group allows for the covalent coupling of DBCO-dsDNA-biotin tethers and DBCO-ssDNA capture molecules. The particles were attached to the sensing surface via the dsDNA tether molecules. Subsequently, the remaining free streptavidin binding sites on the particles were blocked with biotin-PEG to prevent multitethering and minimize nonspecific interactions between particles and the sensing surface. Finally, the DBCO-ssDNA capture molecules were hybridized with an ssDNA-solanidine conjugate, an analyte-analogue molecule that enables the formation of specific, reversible bonds between antibodies on the particles and analogues on the sensing surface. Due to the reversible nature of the antibody–analogue interactions, the particles switch between bound and unbound states. In the presence of analyte molecules, the analyte molecules can bind to the antibodies, thereby changing the switching rate of the particles. In a competitive t-BPM sensor, the switching signal is inversely proportional to the analyte concentration. At low analyte concentrations, particles frequently switch between bound and unbound states, as analyte molecules do not occupy the binding sites on antibodies. Conversely, at high analyte concentrations, the analyte molecules occupy the antibody binding sites, reducing the frequency of switching events.^20,27^

In a t-BPM sensor, thousands of particles are simultaneously tracked using bright-field video microscopy and an algorithm detects the transitions of particles between bound and unbound states.^28^ The algorithm identifies switching events in the individual time traces, enabling the calculation of the average number of switching events per particle per unit of time.^29^ The resulting switching rate is reported as the activity signal, measured in millihertz (mHz).

The competitive GA t-BPM sensor has been proven to detect both α-solanine and α-chaconine, the dominant GAs in potatoes.^13^ Figure 1B shows the continuous monitoring of GAs in diluted potato fruit juice (PFJ) samples, through a series of 10 dose–response curves (DRCs) measured on a single t-BPM sensor over approximately 15 h. The top panel displays the applied concentration–time profile, with GA concentrations ranging from 0.24 to 8.16 μM. The bottom panel shows that the sensor signal depends on the analyte concentration, with increasing GAs concentrations leading to lower switching activity values.

During the continuous monitoring of GAs in diluted PFJ samples, shown in Figure 1B, the maximum signals observed at 0 μM analyte concentration gradually decreased from 75 mHz to about 34 mHz over the measured period. To shed light on the molecular origins of such long-term signal changes, we conducted t-BPM experiments under static conditions, without any analyte solution flushed into the chamber and without any fluid replacement, over extended periods, as previously done for a competitive cortisol t-BPM sensor.^25^ After activating the sensor with analogue solution, the fluidic chamber was flushed with PBS containing 0.5 M NaCl to remove unhybridized analogue molecules. Subsequently, the sensor signal was continuously recorded for approximately 20 h without any fluid exchange. Figure 1C shows that the switching activity of the sensor exhibits a gradual decrease over time, characterized by a double exponential behavior: a fast decrease in the first few hours with a signal loss rate of about 1.35 ± 0.05 per hour, followed by a slower decrease with a signal loss rate of about 0.10 ± 0.01 per hour.

Figure 1D proposes several mechanisms that might underly the observed fast and slow signal changes in the t-BPM sensor, including the dissociation or functionality loss of specific binders (antibodies and/or analogues), gradual loss of particles, nonspecific interactions, multivalent binding of particles, or other mechanisms. We conducted a series of experiments to elucidate the molecular origins of the fast and slow signal changes. The investigations include single-sided aging and equilibrium shift experiments to study long-term signal changes (Figure 2), analysis of motion patterns and particle populations to investigate both fast and slow signal changes (Figure 3), and experiments with molecular blocking strategies (Figure 4).

**Figure 2:**
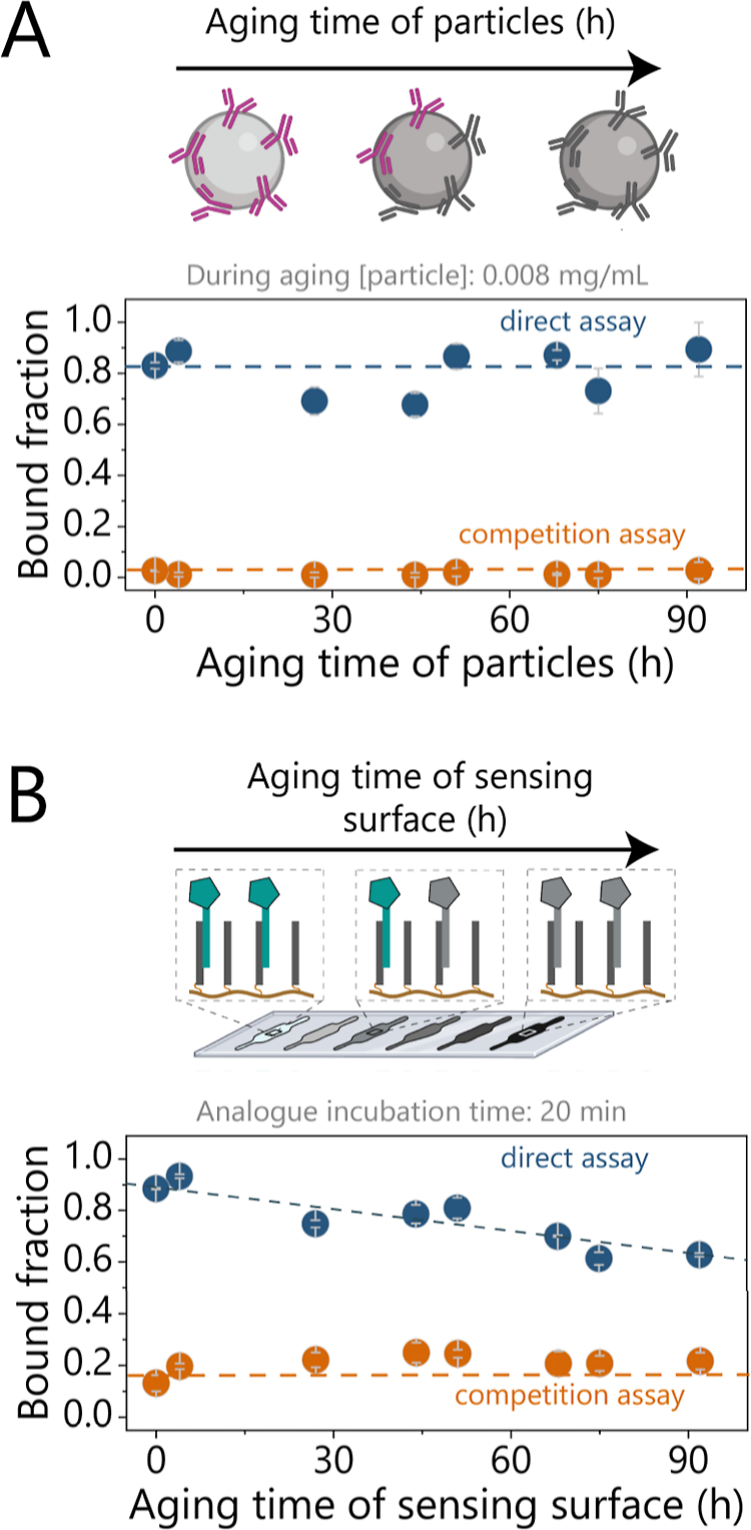
Single-sided aging experiments, with both direct and competition assay readouts, employing free particles on a sensing surface (f-BPM). The f-BPM experiments were performed on cyclic olefin copolymer microscopy slides, each having eight individual fluidic channels. (A) Streptavidin-coated particles functionalized with *anti*-solanidine antibodies were prepared at different time points over 4 days. The particles were diluted 1:1200 in PBS containing 0.5 M NaCl and aged at RT while rotating. On the last day, the aged particle solutions were further diluted to 1:2500 in buffer (for the direct assay, blue) or in buffer containing 16 μM of GA (for the competition assay, orange), achieving a final concentration of 0.008 mg/mL. The solutions were then added to sensing surfaces, freshly functionalized with 10 nM ssDNA-solanidine analogue. (B) Sensing surfaces functionalized with ssDNA-solanidine analogue molecules were prepared at different time points over 4 days. Flow cells were incubated with 10 nM ssDNA-solanidine analogue solution for 20 min. Then, the analogues were washed away, and the functionalized sensing surfaces were stored in PBS containing 0.5 M NaCl at RT for aging. To prevent the fluidic channels from drying, the flow cells were aged in a humidity chamber. On the last day, the prepared sensing surfaces were tested using freshly prepared particle solution (0.008 mg/mL, 2500 times dilution) suspended either in buffer without GA (direct assay) or in buffer with 16 μM GA (competition assay). Dashed lines in the plots are guides for the eye.

**Figure 3:**
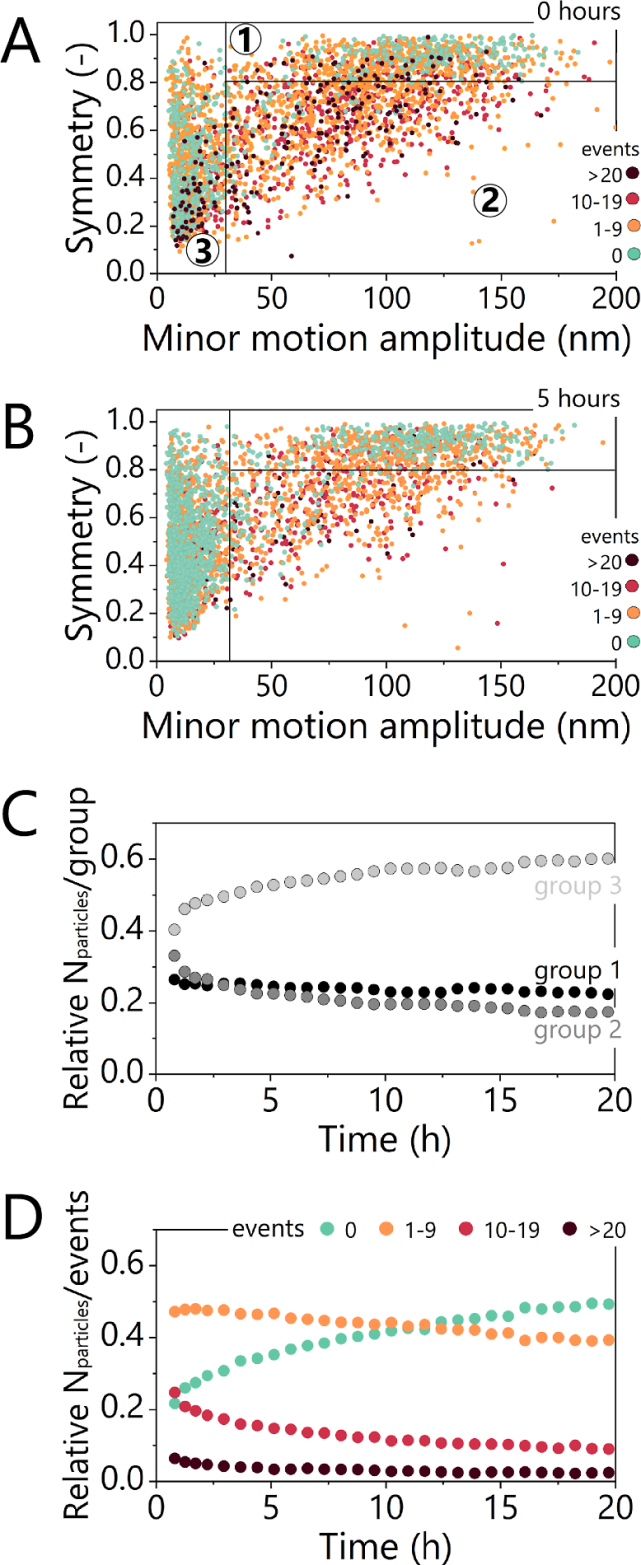
Analysis of particle motion patterns and switching activity in a competitive t-BPM GA sensor over 20 h. Particle motion distribution graphs are plotted as a function of time for the same sensor presented in Figure 1C, with no analyte present in solution and no fluid exchange (static mode flow cell), observed at 0 h (A) and 5 h (B). (A) The graph shows the distribution of motion pattern properties of the individual particles in the competitive t-BPM sensor, with each data point representing a single particle. The graph is divided into three groups based on minor motion amplitude and symmetry in order to analyze the particle behavior over time.^25^ The color of a dot indicates the number of switching events of that particle. The method used to construct these distribution plots is provided in the Supporting Information Section S4. (C) The relative number of particles classified into the three motion pattern groups plotted over time, based on data from Figure 1C. (D) The relative number of particles categorized by the number of switching events per particle (0, 1–9, 10–19, or more than 20 events) plotted over time, as derived from the data in Figure 1C.

**Figure 4:**
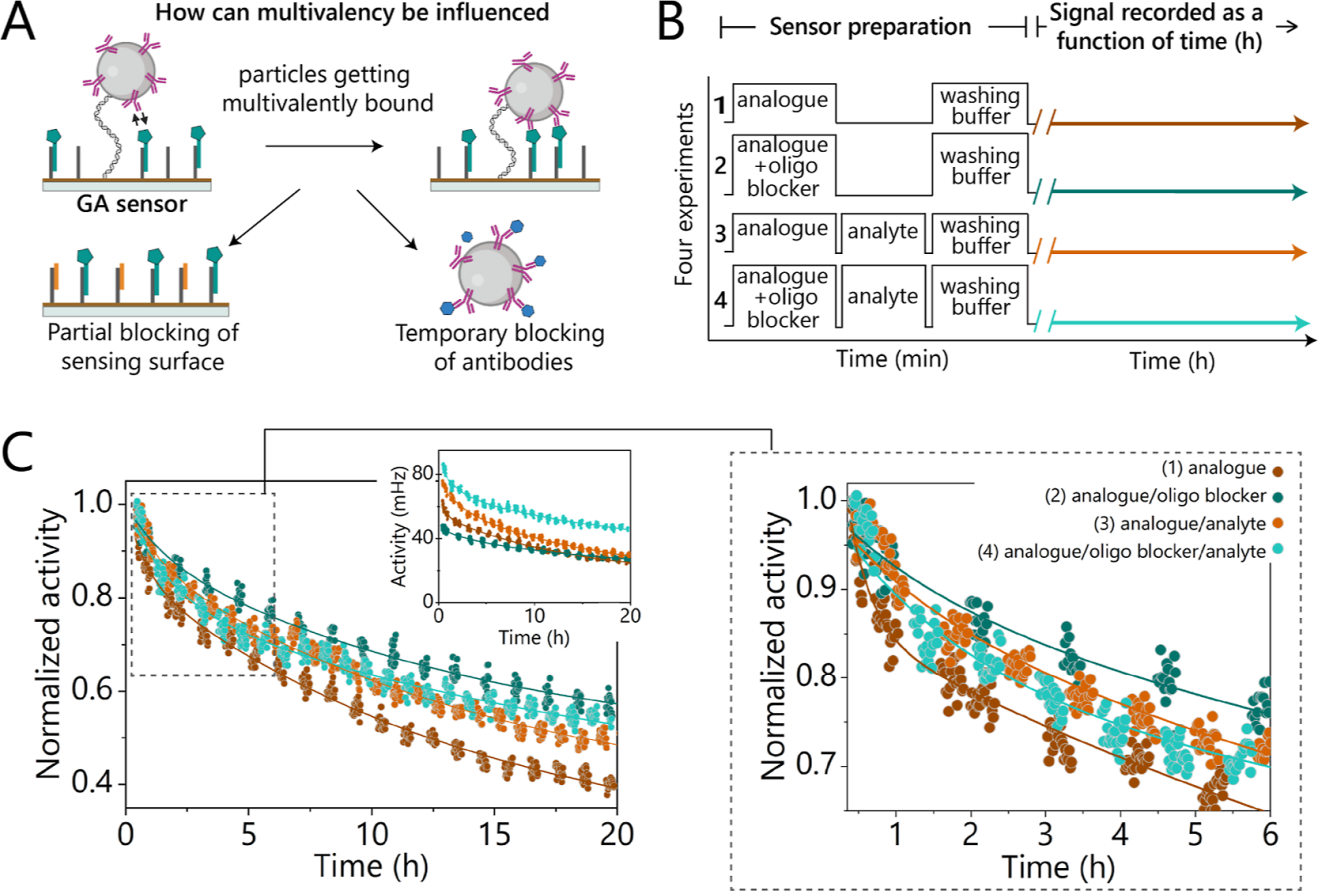
Investigation of the molecular mechanism underlying fast signal changes in the competitive GA sensor, conducted in a static mode, without analyte in solution and without fluid exchange. (A) Proposed blocking experiments. Left: partial blocking of the sensing surface using an oligo blocker, i.e. ssDNA without solanidine, hybridized to the capture oligo. Right: temporary blocking of antibodies on the particles by adding a high concentration of analyte. (B) Experiments conducted in separate flow cells to investigate the mechanisms behind fast signal changes in the competitive GA sensor. Each experiment started with different preparation steps, followed by a 20 h measurement of particle switching activity. Experiment 1: The fluidic chamber was provided with analogue solution, followed by a buffer wash to remove any unhybridized analogue molecules. Experiment 2: A mixture of analogue and oligo blocker solution was flown into the chamber, followed by a washing step to remove unhybridized analogue and oligo blocker. Experiment 3: The chamber was first provided with analogue solution, then flushed with a solution containing a high concentration of analyte, followed by a buffer wash to remove the analyte molecules present in the chamber. Experiment 4: A mixture of analogue and oligo blocker solution was flown into the chamber, followed by the addition of a high concentration of analyte solution, and then washed to remove the analyte molecules present in the chamber. (C) Sensor signals were recorded over approximately 20 h following the experimental preparations detailed in panel (B). Each data point represents a 1 min measurement period. A zoom-in of the first 6 h is shown on the right. Activity signals from different sensor preparations were normalized to evaluate the effects of each experimental condition. The maximum signals observed after analogue addition were set as the baseline (normalized to 1), and all subsequent activity values were scaled accordingly. Normalized curves were fitted using a double-exponential function: *y* = *A*_0_ + *A*_1_·exp­(−*k*_1_·*x*) + *A*_2_·exp­(−*k*_2_·*x*), with amplitude values, *A*_0_, *A*_1_, and *A*_2_ restricted to 1. Fit parameters are provided in Table S1 (Supporting Information Section S11). Duplicate measurements are provided in Supporting Information Section S11.

### Single-Sided Aging Experiments

To differentiate between changes occurring due to the particles and changes due to the sensing surface, we used single-sided aging experiments wherein only one of the components (particles or surface) was aged with variable durations.^20^ The aged component was tested in a free-BPM assay (f-BPM), where particles freely move above the functionalized sensing surface under gravitational forces, without the tether molecule being present.^21,25^ In a direct assay, both particles and the sensing surfaces were functionalized with specific quantities of antibodies and analogue molecules, and the bound fraction signal was studied as a function of the aging time of the respective component (particles or surface); see Figure 2. In a competition assay, the aged components were tested with a high concentration of GAs, to investigate the occurrence of nonspecific binding. Before the aging study, we tested the reproducibility of the f-BPM experiments across different flow cells, particle batches, and slides (see Supporting Information section S2). Figure 2A shows the results of the particle aging experiment. Batches of particles were functionalized with antibodies at different time points over 92 h (approximately 4 days) and were suspended in PBS containing 0.5 M NaCl. After aging, the particle batches were tested on freshly prepared sensing surfaces. Figure 2A shows the bound fraction values of the aged particles as a function of the aging time for both direct and competition assay readouts. The results indicate that aging does not affect the specific or nonspecific binding properties of the particles, suggesting that the antibodies on the particles neither dissociate nor lose their binding functionality within the tested aging periods. The stability of the particles is independent of the particle concentration during aging (see Supporting Information Section S3).

Figure 2B illustrates the results of the surface aging experiment. Sensing surfaces were prepared on the same day but functionalized with analogue molecules at different time points by hybridizing ssDNA-solanidine conjugates for 20 min, followed by a washing step with PBS containing 0.5 M NaCl to remove any unhybridized analogue molecules. Functionalized surfaces were aged at RT for different durations, up to 92 h, with flow cells stored in a humidity chamber to prevent fluid evaporation. After the aging process, the aged sensing surfaces were evaluated using both direct and competition f-BPM assay readout, employing a freshly prepared batch of particles at a concentration of 0.008 mg/mL. The bottom panel of Figure 2B presents the bound fraction values for the aged sensing surfaces. The competition assay results show low bound fraction values for all aging durations. In contrast, the bound fraction values in the direct assay readout exhibit a gradual decrease with increasing aging time. In a surface aging experiment with analogue molecules present in solution (see Figure S2B), the bound fraction values are constant as a function of the aging time. These results indicate that the gradual decrease in the bound fraction observed in Figure 2B for the direct assay readout is primarily due to the dissociation of ssDNA-solanidine conjugates from the sensing surface as a function of the aging time.

### Distributions of Particle Motion Patterns and Switching Events

BPM sensors are composed of thousands of particles, with each particle functioning as an individual sensor. The switching activity in the BPM sensor reflects the average response of all tracked particles. Due to the heterogeneous distribution of binders on particles and surfaces, individual particles contribute differently to the overall signal.^30,31^ Figure 3A presents a distribution plot of particle motion patterns for the GA t-BPM sensor of Figure 1C. Particles are plotted according to their motion amplitudes along the major (*A*_major_) and minor (*A*_minor_) motion axes; see Supporting Information Section S4. These parameters are derived from the covariance matrix of the position data and characterize the particle motion pattern. The symmetry of the motion pattern, calculated as the ratio between the minor and major motion axes (*A*_minor_/*A*_major_), is plotted on the *y*-axis, ranging from 0 to 1. The *x*-axis represents the minor motion amplitude, ranging from 10 to 200 nm. Based on these parameters, particles with high rotational symmetry (symmetry >0.8) and minor motion amplitude >30 nm are assigned to group 1, representing single-tethered particles that exhibit disk-like motion patterns. Particles with significant motion amplitude (>30 nm) and symmetry <0.8 are assigned to group 2, corresponding to particles that interact dynamically with the sensing surface, so they have heterogeneous motion patterns as a result of the switching behavior. Particles with restricted motion patterns (minor motion amplitudes <30 nm) are classified into group 3, corresponding to stuck particles, due to multivalent and/or nonspecific interactions.^19,25^

In addition to classifying particles by motion patterns, Figure 3A further categorizes them by their switching activity, using distinct colors to represent different switching frequencies. The plot shows that group 2 contains very few nonswitching particles (green dots), as this group primarily consists of particles that actively switch between bound and unbound states. In contrast, groups 1 and 3 have a higher population of nonswitching particles and a lower number of switching particles compared to group 2.

Figure 3A and B show how the distributions depend on the aging time, specifically at 0 h (Figure 3A) and 5 h (Figure 3B), for the sensor depicted in Figure 1C. Changes in the distribution of particle populations over 0–5 h correspond to the fast signal changes observed in the GA t-BPM sensor. Differences on that time scale are a decrease in the number of switching particles in group 2 and an increase in nonswitching particles in group 3. The increase in nonswitching particles is particularly pronounced in group 3, indicating that the switching particles transition to a nonswitching state due to multivalent binding or nonspecific interactions with the sensing surface. Distribution plots illustrating the particle behavior for aging times beyond 5 h are provided in Supporting Information Section S5.

Figure 3C quantifies particle populations across all three groups as a function of the sensor aging time. Initially, particles are distributed across the groups as follows: 28% in group 1, 33% in group 2, and 40% in group 3. However, after 20 h, a significant shift in distribution is visible. Group 1 decreased by 18%, group 2 decreased by 45%, while group 3 increased by approximately 50%. The increase in group 3 is opposite to the time-dependent activity observed in Figure 1C. During the first few hours of particle tracking, group 3 exhibited a fast increase in particle population, followed by a slower increase over the next 15 h. Specifically, the fast increase resulted in a 35% rise in group 3 within the first 5 h, followed by an approximate 11% increase over the next 15 h.

Figure 3D illustrates the distribution of the particle populations based on their switching activity. The data reveal that the number of switching particles decreases with aging time while the population of nonswitching particles increases considerably. Notably, the increase in nonswitching particles follows a similar trend to the group 3 population increase observed in Figure 3C. Within the first 5 h, nonswitching particles increased rapidly by approximately 67%, correlated with fast signal changes, followed by a more gradual increase of about 42% over the following 15 h, associated with slow signal changes. Conversely, the populations of both medium and highly switching particles exhibited a decrease of approximately 19% and 64%, respectively, over the 20 h of the particle tracking period. The trends observed in the motion groups and switching activity classifications suggest that the fast changes in switching activity may be influenced by a combination of nonspecific interactions and particles becoming multivalently bound to the sensing surface. To further investigate this hypothesis, Supporting Information Section S6 presents the analysis of nonswitching particles across different groups. Figure S5 shows that the largest population of nonswitching particles corresponds to group 3, which significantly increases within the initial hours of signal tracking and continues to grow over time. In contrast, the increase of nonswitching particles in group 1, linked to the gradual loss of binders and associated with slow signal changes, was relatively small throughout the measurement period. To evaluate the contribution of nonspecific interactions to the signal decrease over time, Supporting Information Section S7 examines particle distributions before and after analogue addition, as well as post-analyte addition. Before analogue addition, most particles are single-tethered.

Analogue addition induces reversible switching interactions, evidenced by shifts in particle motion distribution and an increased number of switching events. Subsequent analyte addition determines the interaction specificity. Figure S6 shows that while most interactions were reversed upon analyte addition, a small fraction of particles did not return to their pre-analogue motion state, indicating some persistent nonspecific interactions. See Supporting Information Section S7 for further details.

Since most particles are in group 1 before analogue addition and return to this group after analyte addition, it is hypothesized that the fast signal loss is primarily driven by particles becoming multivalently bound after the addition of analogue molecules. This multivalent binding restricts the ability of particles to switch between bound and unbound states, reducing the number of switching events and leading to signal loss. This hypothesis is further supported by the observed increase in unbound state lifetimes as a function of aging time. Specifically, unbound state lifetimes shifted toward longer durations, likely due to the reduced availability of binders, see Supporting Information Section S8.

### Blocking Strategies for Investigating Changes in the GA t-BPM Sensor

The analysis of particle distributions in the GA t-BPM sensor revealed that the changes in activity signal arise from two distinct mechanisms. Slow signal changes are due to a gradual loss of affinity binders over time, while fast signal changes are largely attributed to particles becoming multivalently bound to the sensing surface. One potential hypothesis for the observed fast signal changes was that they were caused by residual analogue molecules remaining in the measurement chamber due to an incomplete fluid exchange during the washing step. However, this hypothesis was refuted through control experiments, which demonstrated a double exponential signal loss for the sensor with sequential flushing of a buffer. Detailed information on these controls can be found in Supporting Information Section S9.

Figure 4A proposes two strategies to further investigate the origins of the fast signal changes in the GA t-BPM sensor, by reducing the ability of the particles to form multivalent bonds: (1) Partial blocking of the ssDNA capture molecules on the sensing surface with a complementary ssDNA oligo blocker, and (2) temporary blocking of antibodies on particles by adding a high concentration of analyte molecules. Figure 4B illustrates four sensor preparation procedures designed to compare the blocking strategies outlined in Figure 4A. The sensor was prepared by an activation step with either analogue molecules only (experiments 1 and 3), or with a mixture of analogue molecules and oligo blocker (experiments 2 and 4). Supporting Information Section S10 details control experiments to evaluate the effectiveness of the oligo blocker and explains the reason for its simultaneous addition with the analogue molecules. In experiments 3 and 4, the analogue step was followed by a high concentration of GA. The idea of these experiments was to temporarily block the binding of analogue molecules to antibodies on the particles to investigate if a two-step process (analogue molecules first binding to antibodies, and thereafter hybridizing to the sensing surface) might play a role in the formation of multivalent bonds between particle and surface. In all cases, the sensor preparations were ended by a final buffer wash to clean the measurement chamber. After the sensor preparations, the sensor activity signals were measured over 20 h, see Figure 4C, with a zoom-in of the first 6 h.

Experiments 1 and 2 focus on the effect of using an oligo blocker. Compared to experiment 1, the sensor in experiment 2 showed significantly less signal loss during the first 6 h. This is also shown in the particle motion analysis, which shows that the fraction of particles migrating to group 3 is less in the first 6 h (see Supporting Information Section S12). We hypothesize that the oligo blocker reduces the probability that particles become multivalently bound to the sensing surface after the activation process. Given the high density of capture molecules on the sensing surface,^31^ multivalency is hypothesized to arise from high densities of analogue molecules near the particles. The oligo blocker reduces the analogue density, thereby lowering the probability of multivalent binding. Experiment 3 also showed a decrease in fast signal changes compared to experiment 1, which might be attributed to the prevention of a two-step binding process. It is hypothesized that analogue molecules can initially bind to antibodies on the particles and, thereafter hybridize to the surface. The addition of analyte in the solution might displace the particle-bound analogue molecules and reduce the probability that they hybridize to the surface. The displaced analogue molecules are then removed during the washing step, reducing the chance that particles become multivalently bound. Experiment 4, which combined both blocking strategies, demonstrated a similar decrease in fast signal changes as observed in experiments 2 and 3 (see the signal loss rates in Supporting Information Section S11). These results show that molecular blocking strategies, on the sensing surface and on the particles, can influence the fast signal changes in the competitive GA t-BPM sensor. Further analysis of their effects on particle distributions over time is provided in Supporting Information Section S12.

## Discussion and Conclusions

We studied gradual signal changes in a competitive t-BPM GA sensor over tens of hours, in conditions without analyte and without flow. The results show both fast and slow signal changes, observed on time scales of 0–5 h and 5–20 h, respectively. Experiments with single-sided aging and motion pattern analysis show that the slow signal changes relate to a gradual loss of analogue molecules from the sensing surface, while the properties of the antibody-functionalized particles are stable. The fast signal changes dominantly relate to the formation of multivalent bonds between particles and the sensing surface.

Previously, we studied signal changes in a cortisol t-BPM sensor, which revealed slow signal changes but not the fast signal changes. The GA and cortisol sensors have many common elements, such as the use of a PLL-*g*-PEG polymer layer on the substrate, analogue-ssDNA conjugate molecules hybridized to the PLL-*g*-PEG layer, dsDNA tether molecules between particle and surface, and biotinylated antibodies coupled to streptavidin-coated particles. However, several components are not the same: the antibodies are different (*anti*-GA antibodies vs *anti*-cortisol antibodies), the analogue molecules are different (ssDNA-solanidine conjugate vs ssDNA-cortisol conjugate), and the substrate materials are different (cyclic olefin copolymer vs polyethylene derivative). We hypothesize that the effective densities of binder molecules on particles and/or surface are higher in the GA sensor than in the cortisol sensor, increasing the probability that multivalent bonds are formed. Concerning functional properties over tens of hours, the GA and cortisol sensors both show a gradual loss of signal and a loss of analogue molecules. A clear difference between the sensors is that the antibody-functionalization of the particles in the GA sensor is more stable than in the cortisol sensor.

The experiments with blocking steps (Figure 4) revealed a slowing down of the fast signal changes. Blocking of the sensing surface and/or the particles during the preparation of the sensor reduces the signal changes, presumably due to a lower probability that particles form multivalent bonds with the sensing surface. Further research will be needed to determine the precise underlying mechanisms, e.g. possible pathway changes within the functionalization process and changes in the resulting distribution of analogue molecules on the sensing surface.

Further research can help to understand the molecular mechanisms in the sensor and increase the operational lifetime of the t-BPM sensor. Given the demonstrated stability of the antibody-functionalized particles, the focus of further work should be on the sensing surface, e.g. improvements of the coupling methodology of the analogue molecules. We envision that advancements in this area will lead to the development of robust sensors suitable for continuous monitoring over prolonged periods.

## Supplementary Material



## Notes

Materials and methods, BPM assay reproducibility, aging and equilibrium-shift conditions, BPM lifetime analysis, analysis of nonswitching particles over time, particle distribution over time with blocking strategies (PDF)
