# Supplementary material for: Understanding Fast and Slow Signal Changes in a Competitive Particle-Based Continuous Biosensor
Source: Anal Chem. 2025 Jun 10;97(24):12719–27. doi: 10.1021/acs.analchem.5c01457 (PMC12199230; doi:10.1021/acs.analchem.5c01457)
Supplement: Supplementary file 1 [file ac5c01457_si_001.pdf]

Understanding fast and slow signal changes in a competitive particle-based continuous biosensor

Sebastian Cajigas[a], Arthur M. de Jong[b,c], Junhong Yan[d], Menno W.J. Prins[a,b,c,d*]

[a] Department of Biomedical Engineering, Eindhoven University of Technology, 5612 AZ, Eindhoven, The Netherlands
[b] Department of Applied Physics, Eindhoven University of Technology, 5612 AZ, Eindhoven, The Netherlands
[c] Institute for Complex Molecular Systems (ICMS), Eindhoven University of Technology, 5612 AZ, Eindhoven, The Netherlands
[d] Helia Biomonitoring, 5612 AR, Eindhoven, The Netherlands
*Corresponding author: m.w.j.prins@tue.nl

Supporting information

Table of Contents

S1. Materials and methods S2
S2. Study of reproducibility in free-BPM format S3
S3. Aging experiments with equilibrium-shift conditions S4
S4. Motion patterns of tethered particles before analogue addition S5
S5. Motion patterns of tethered particles as a function of time S6
S6. Non-switching particles as a function of time S7
S7. Specific and nonspecific interactions in the t-BPM sensor S8
S8. Analysis of state lifetimes without analyte and fluid exchange S10
S9. Sequential buffer flushing to remove leftover analogue molecules S11
S10. Effectiveness of oligo blocker S12
S11. Duplicate measurements of partial blocking strategies S14
S12. Distribution of particles for sensor with blocking strategies S16
References S18

S1. Materials and methods

Surface functionalization for t-BPM. Cyclic olefin copolymer (COC) injection-molded cartridges (Axxicon) were cleaned by 10 min sonication in Milli-Q water and dried with a nitrogen stream. Afterward, the cartridges were placed in a UV ozone cleaner for 30 min and then sealed with optically transparent tape (#232702 Sealing tape, Thermo Scientific). Thereafter, the cartridges were filled with 25 µL of polymer solution containing 0.45 mg/mL poly(l-lysine)-grafted poly (ethylene glycol) (PLL-g-PEG, SuSoS) and 0.05 mg/mL azide functionalized PLL-g-PEG (azide-PLL-g-PEG, Nanosoft Biotechnology LLC) and incubated for 3 hours in a humidity chamber. Then, the polymer solution was replaced with 25 µL of solution containing 0.4 nM DBCO-dsDNA-biotin tether (221 bp dsDNA) and 3 µM DBCO-ssDNA capture oligo (DBCO – 5' – GTG CGG CAG GGG TAA GAC CA -3') and incubated for at least 3 days.

Particle functionalization for t-BPM. 4 µL streptavidin-coated magnetic particles (10 mg/mL, Dynabeads MyOne Streptavidin C1, 65001, Thermo Scientific) were mixed with 4 µL 250 nM biotinylated anti-solanidine antibody (prepared as described in Vu et al13) and incubated for 30 min at room temperature (RT) while rotating. Subsequently, 3 µL of 10 µM polyT-biotin (biotin- 5'- TTT TTT TTT TTT TTT T – 3') and 10 µL PBS were sequentially added to the particle mixture and incubated for 45 min. After incubation, functionalized particles were washed twice with PBS containing 0.05% Tween-20 (PBST) and reconstituted in 300 µL of 0.5 M NaCl in PBS. Lastly, the suspended particles were sonicated in a sonication bath for 30 s to disaggregate particle clusters before use.

t-BPM sensor assembly. On the measurement day, a COC cartridge was placed on a homemade compact microscope setup. The cartridge was connected to a microfluidic rotary valve (10 ports valve, LSPone, Advanced Microfluidics) on one end and to a laboratory Programmable Syringe Pump (8 ports valve, LSPone, Advanced Microfluidics) on the other end using flangeless PFA fittings and ETFE ferrules (1/4"-28 to 1/16" OD, Darwin Microfluidics) and rigid tubing. 400 µL of *anti*-solanidine antibody-functionalized particles were flown into the functionalized COC cartridge at a speed of 100 µL/min. The particle solution was incubated for 10 min to allow the particles to sediment onto the sensing surface and become tethered. Subsequently, 200 µL of 100 µM of 1 kDa mPEG-biotin (PG1-BN-1k, Nanocs; blocking solution) was flown through the cartridge and incubated for 20 min to block the remaining free streptavidin molecules on the particles. The sensor was activated by flowing 100 µL of 10 nM solanidine-ssDNA analogue solution (prepared as described in Vu *et al*[13]) and incubated for around 5-10 min. The excess analogue was removed by flowing 200 µL of 0.5 M NaCl in PBS through the cartridge.

Single-sided aging experiments on f-BPM.

Cyclic olefin copolymer (COC) microscopy slides (Chipshop, 10000002, 1.0 mm) were used to individually study the aging of particles and sensing surfaces in the f-BPM format. The COC slides were cleaned by 10 min sonication in Milli-Q water, followed by drying with a nitrogen stream. Subsequently, the slides were exposed to UV ozone for 30 min. Thereafter, custom-made flow cell stickers were attached to the COC slides. The COC slides were functionalized as described in the t-BPM section. Particles were functionalized as described in the t-BPM section, with modified blocking after the antibody functionalization: free streptavidin binding sites were blocked by sequentially adding 10 µL of 10 µM polyT-biotin and 10 µL of PBS.

f-BPM particle imaging and data analysis. Single-sided aging measurements with f-BPM readout was performed in a homemade compact microscope setup with a motorized XY stage (ASR series, 100 mm × 120 mm travel; Zaber Technologies Inc.) using brightfield microscopy, a green LED light source, and 10x magnification (10x DIN Achromatic Finite Intl Standard Objective, Edmund Optics). Particle motion was recorded for 0.5-1-min blocks with a frame rate of 60 Hz and 3 ms exposure time using a high-speed camera (FLIR Blackfly S BFS-U3-31S4M) with a field of view of 0.53 × 0.71 mm^2. The recorded frames from each measurement were analyzed in real-time using a specialized particle tracking software as detailed by Bergkamp *et. al*[27]. The software enables the differentiation between bound and unbound states based on diffusivity time traces derived from the recorded particle video data.

t-BPM particle imaging and data analysis. In the competitive t-BPM GA experiments, particles were tracked before, during, and after surface activation with analogue molecules (solanidine-ssDNA), as well as after each added analyte concentration. The particles were imaged using a homemade compact microscope setup, employing brightfield microscopy with 10x magnification (10x DIN Achromatic Finite Intl Standard Objective, Edmund Optics) and a green LED light source. The particle motion was recorded with a high-speed camera (FLIR Blackfly S BFS-U3-31S4M) with a field of view of 0.53 × 0.71 mm^2. Data acquisition was conducted in 1-min blocks with a frame rate of 30 Hz and an exposure time of 0.15 ms. The microscope setup was controlled with a Matlab program, and particle positions were tracked in real-time using particle tracking software described by Bergkamp *et. al*[27]. Long-term signal changes of the t-BPM GA sensor were analyzed in terms of activity, lifetimes, and motion patterns. Minor motion amplitude and symmetry were employed to construct motion pattern distribution maps of the tethered particles.

S2. Study of reproducibility in free-BPM format

The f-BPM sensing format used for the single-sided aging and equilibrium shift experiments presented in Figure 2 was tested for reproducibility[1]. Concentrations of 250 nM of anti-solanidine antibody and 10 nM of ssDNA-solanidine conjugate (analogue) were used to functionalize particles and the sensing surface, targeting a bound fraction of approximately 80%. These conditions were chosen to enable the observation of both increases and decreases in the bound fraction as a function of the aging time.

Reproducibility was evaluated by conducting experiments with different batches of particles, flow cells, and slides, see Figure S1. Across all tested conditions, the f-BPM format assay showed bound fraction values of about 80%, confirming the reproducibility of the experimental setup.

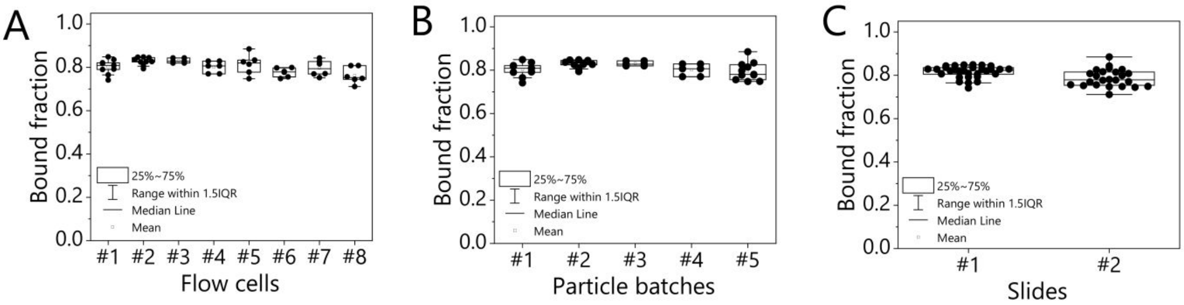

Figure S1. Reproducibility study of free-BPM (f-BPM) assay format. (A) Eight separate flow cells were employed to evaluate the reproducibility of the f-BPM sensor. (B) Five independently prepared particle batches were measured across these eight separate flow cells. (C) Two microscopy COC slides, each with four flow cells on top, were measured on two consecutive days. To ensure homogeneity within the flow cells, measurements were taken at different points across each flow cell on the two different slides and for all five batches of particles.

S3. Aging experiments with equilibrium-shift conditions

Figure 2 in the main text depicts the results of single-sided aging experiments for both particle and the sensing surface. Figure 2A shows the aging of particles at a concentration of 0.008 mg/mL. Figure 2B presents the aging of the sensing surface, where the analogue solution was incubated for 20 min and then replaced with a buffer solution (without analogue) in the measurement chamber.

To differentiate between denaturation processes (e.g. loss of binding functionality of antibodies or analogues) and potential dissociation processes (such as antibody dissociation from particles or analogue dissociation from the sensing surface), equilibrium-shift experiments were studied[1]. Figures S2A presents particle aging experiment at a higher particle concentration of 0.07 mg/mL. On the day of measurement, the aged particles were further diluted to 0.008 mg/mL. The results in Figure S2A show a stable bound fraction over the aging period. Consistent with Figure 2A, the aging process did not result in a significant loss of particle functionality, as both the direct-assay and competition-assay readouts remained stable throughout the aging period.

Figure S2B shows an equilibrium-shift experiment on the sensing surface, where the analogue solution was not replaced after 20 min of incubation, unlike the procedure in Figure 2B. During the aging period, the fluidic chambers contained analogue molecules at a concentration of 10 nM. The data indicate that the bound fraction values, with both the direct and competition assay readout, remained constant as a function of the aging time. This suggest that the decrease in bound fraction observed in Figure 2B results from the dissociation of analogue molecules, rather than a loss of functionality of analogue molecules hybridized on the sensing surface.

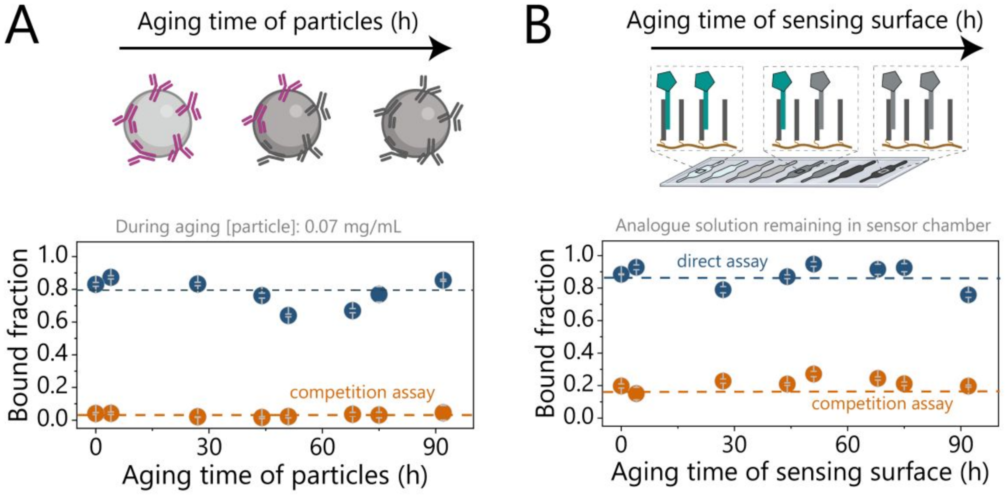

Figure S2. Equilibrium-shift studies on particle and sensing surface. (A) Particles functionalized with *anti*-solanidine antibody were diluted 150-fold to a concentration of 0.07 mg/mL in 0.5 M NaCl in PBS and were aged over different periods ranging from 0 to 92 hours while rotating at room temperature. The aged particles were then further diluted 2500-fold to a concentration of 0.008 mg/mL and measured on freshly prepared sensing surfaces with 10 nM of analogue using both direct and competition assay readouts. (B) Sensing surfaces functionalized with ssDNA-solanidine conjugates were prepared by adding 10 nM of analogue on different days over 4 days. The analogue solution was not replace with a buffer solution after 20 min of incubation, thus, the flow cells remained in continuous incubation with the analogue solution until the measurement day. On the final day, all the aged sensing surfaces were washed with 0.5 M NaCl in PBS and then studied with freshly *anti*-solanidine antibody-functionalized particles. Measurements were performed using both direct and competition assay readout. Dashed lines in the figures serve as guides for the eye. Schematic illustrations were created using Biorender.com.

S4. Motion patterns of tethered particles before analogue addition

Figure 3 in the main text shows the distribution of particle motion in a competitive GA t-BPM sensor under static conditions, where no analyte is present in solution and without fluid exchange into the fluidic chamber.

Here, we focus on the distribution characteristics of a GA t-BPM sensor without analogues on the sensing surface, see Figure S3A. The tethered particles were functionalized with *anti*-solanidine antibodies; however, the sensing surface did not contain analogue molecules. This lack of analogue molecules allowed the particles to move freely within the range permitted by the tether molecule.

The motion distribution map of tethered particles is categorized into three distinct groups based on the motion patterns, as explained in the main text[2]. The bottom panel of Figure S3 illustrates the three characteristic motion patterns of tethered particles, which vary depending on their interaction with the sensing surface. Group 1: disk-like motion patterns are characteristic of particles attached to the sensing surface by a single tether molecule without forming additional bonds with the surface. Group 2: heterogenous patterns are predominantly observed in the presence of analogue molecules, resulting from transient binding between particles and the sensing surface. Group 3: confined patterns occur when particles become stuck due to multivalent binding or nonspecific interactions, leading to a highly restricted motion pattern[1].

Figure S3A top panel reveals that over 70% of the tethered particles are located in group 1, which corresponds to single-tethered particles. About 15% of the particles are categorized in group 3, indicating that they are stuck due to either multiple tethering or non-specific interactions. The remaining particles belong to group 2. Additionally, Figure S3 presents the particle classification using a color scale based on the number of switching events. Most particles are depicted as green dots, indicating zero switching events, which is expected due to the absence of analogue molecules on the sensing surface, as seen in the histogram in Figure S3B. Particles shown with other colors (orange, red, and brown) are associated with non-specific interactions or signal-processing artifacts.

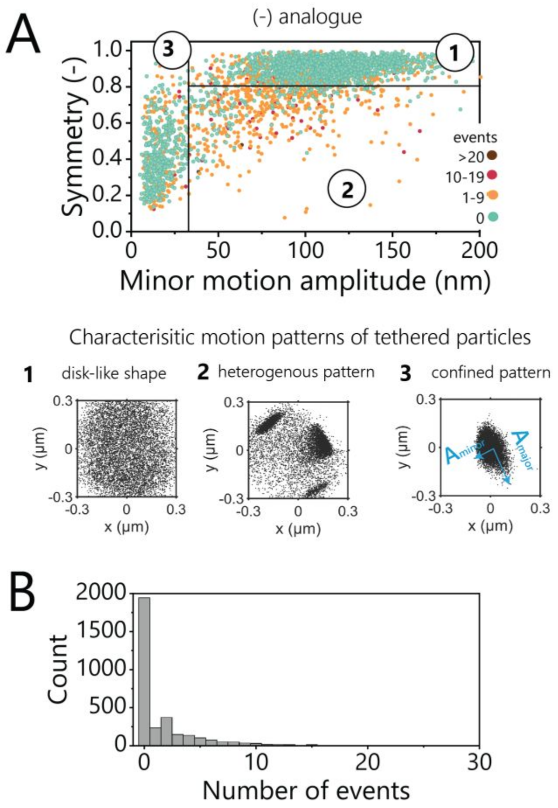

Figure S3. GA t-BPM sensor before the addition of analogue molecules on the sensing surface. (A) The top panel shows the particle motion distribution plot of a GA t-BPM sensor before the analogue addition. The colors represent the number of switching events of each individual particle. The bottom panel depicts characteristic motion patterns of tethered particles depending on their interaction with the sensing surface. (B) Histogram showing the distribution of switching events in the absence of analogue molecules. The average number of switching events at this stage is 1.7, with a corresponding average switching activity of 28 mHz.

S5. Motion patterns of tethered particles as a function of time

Figures 3A and B in the main text depict the motion distribution plot of the GA t-BPM sensor under static conditions at the initial time point (0 hours) and after 5 hours of tracking the particle motion. The data presented in these figures reveal noticeable changes as a function of aging time, particularly the migration of particles from group 2 to group 3, which is associated with fast signal changes in the t-BPM sensor.

Figures S4A, B, and C present motion distribution plots corresponding to aging times of 10, 15, and 20 hours, respectively. These plots show changes in groups 2 and 3. Specifically, there is a decrease in the particle population within group 2, accompanied by an increase in group 3. However, the changes observed after 10 hours are less pronounced compared to those in Figure 3, suggesting a different signal-changing process, potentially related to binder dissociation over time.

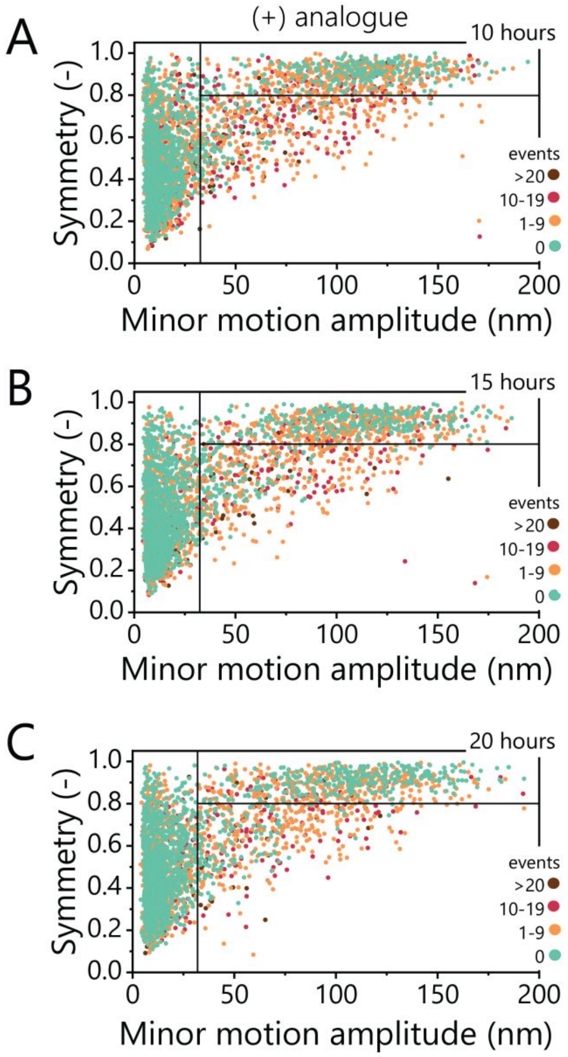

Figure S4. Particle motion distribution plot as a function of time, for the same GA t-BPM sensor presented in Figure 3, observed after (A) 10, (B) 15, and (C) 20 hours.

S6. Non-switching particles as a function of time

Figures 3C and 3D in the main text show how particle populations, categorized into different groups based on characteristic motion patterns and the number of switching events, change over time. Here, we present the distribution of non-switching particles across the different groups.

Figure S5 shows that the increase in non-switching particles (green dots) primarily occurs in group 3, with a low increase observed in group 1. Throughout the entire measurement period, group 2 consistently maintains a very low number of non-switching particles, which is in agreement with the interpretation that this group consists mainly of switching particles. The increase in group 3, representing particles with a confined motion pattern, can be attributed to particles located initially in group 2 (switching particles) that shift to group 3, becoming non-switching particles due to multivalent interactions or some nonspecific interactions, the latter further investigated in Supporting Information 6. The changes observed in group 1 are associated with the migration of particles from group 2 to group 1, likely due to the loss of specific binder molecules, such as antibodies and/or analogues molecules. Additional support is provided by the analysis of state lifetimes, detailed in Supporting Information 7.

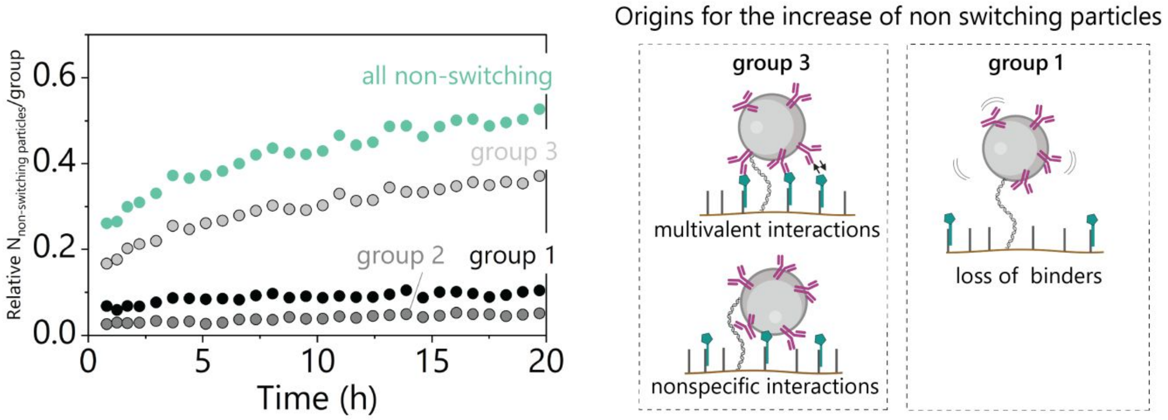

Figure S5. Distribution of non-switching particles over time, categorized in different groups (groups 1, group 2, and group 3) based on their characteristic motion patterns. The data, plotted as a function of time, are derived from the measurements shown in Figure 1C. The right panel schematically illustrates the molecular mechanism underlying the increase in these non-switching particles over time. Schematic illustrations were created using Biorender.com.

S7. Specific and nonspecific interactions in the t-BPM sensor

Figure 3 in the main text illustrates the time-dependent particle distribution across different groups and their switching events. The particle analysis reveals an increase in the particle population in group 3, which mirrors the decrease of switching particles in group 2.

To elucidate the dominant mechanism driving the transition of particles into group 3, Figure S6 examines the particle motion distribution at different sensor preparation steps. This analysis includes evaluating the particle distribution across the different groups and switching events before and after analogue addition, as well as after the addition of a high concentration of analyte.

Figure S6A presents the particle distribution of a GA t-BPM sensor before the addition of the analogue. At this initial stage, the majority of particles are located in group 1, corresponding to single-tethered particles, as expected due to the absence of specific interactions with the sensing surface. This baseline distribution provides an estimated fraction of particle exhibiting non-specific interactions (particles in group 3). Figure S6B shows the particle distribution after the analogue addition. At this step, a significant redistribution occurs, with a decrease in group 1 and increases in groups 2 and 3. This shift indicates that particles are reversibly interacting with the sensing surface, behavior also reflected by the increased presence of switching particles (orange, red, and brown).

Figure S6C displays the particle distribution after the addition of a high analyte concentration (16 μM). The analyte molecules bind to the antibodies on particles, thereby inhibiting their interaction with the sensing surface. In this condition, particles are expected to shift back from groups 2 and 3 to group 1, mirroring their pre-analogue addition state (Fig. S6A). If particles fully return to their initial original state, this would indicate that all switching interactions were specific. Conversely, if some particles persist in exhibiting switching and remain in groups 2 and 3 despite the analyte addition, this would suggest the presence of nonspecific interactions induced by the analogue.

Figure S6D shows the fraction of particles in each group across different sensor preparation steps. Before analogue addition, most of the particles reside in group 1, representing single-tethered particles. Upon analogue addition, group 1 decreases as particles shift to groups 2 and 3. After analyte addition, group 1 largely returns to its initial fraction, but group 3 slightly increases compared to the pre-analogue step, suggesting the increase of nonspecific interactions.

Figure S6E illustrates the particle distribution over the switching events. Before analogue addition, most particles appear green, representing non-switching particles, with a small fraction of low-switching particles (orange). Upon analogue addition, the non-switching particle population (green) decreases significantly, while the switching particle populations (orange, red, and brown) increase. After analyte addition, the non-switching particle population largely returns to its initial fraction, but a small fraction of low-switching particles increases compared to the pre-analogue step, indicating that some particles remained bound despite the presence of the analyte, suggesting an increase in nonspecific interactions. Nonetheless, most particles shifted back to their initial non-switching state, confirming that their interactions with the sensing surface are predominantly specific, with only a small fraction exhibiting nonspecific binding.

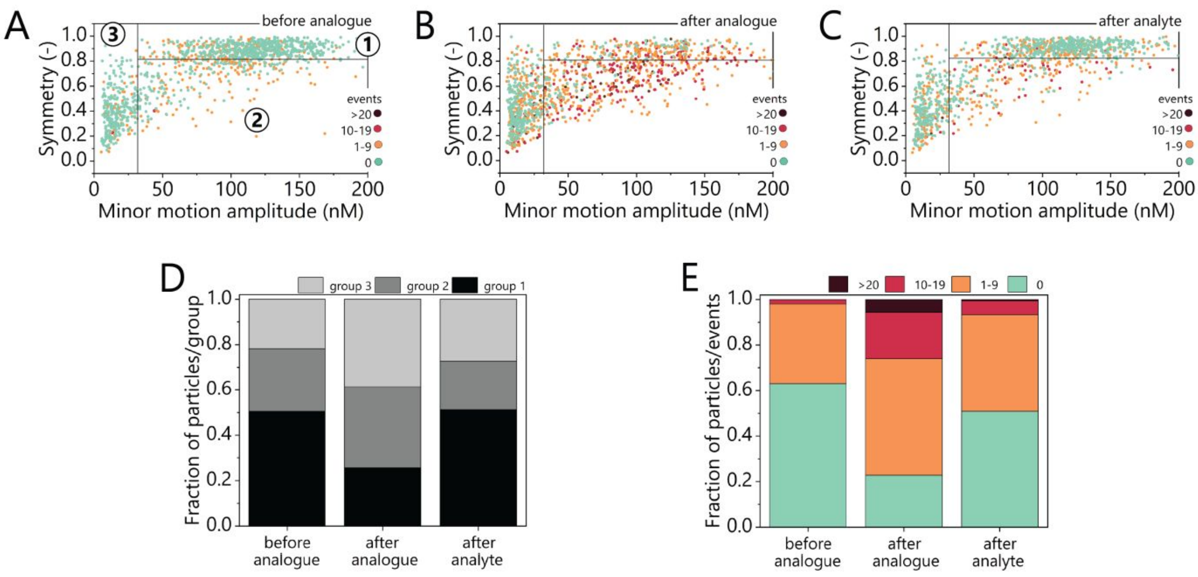

Figure S6. Particle motion distribution as a function of the sensor preparation steps. (A) Motion distribution of tethered particles prior to the addition of the analogue. (B) Particle motion distribution following the analogue addition. (C) Particle motion distribution after the addition of a high analyte concentration (16 µM). (D) Fraction of particles across the three groups for each sensor preparation step. (E) Fraction of particles as a function of their number of switching events observed for each sensor preparation step.

S8. Analysis of state lifetimes without analyte and fluid exchange

BPM is a sensing technology with single-particle and single-molecule resolution, enabling the observation of particle-switching events[3–5]. These events result from transient binding between specific binders on both the particles and the sensing surface[6]. The characteristic times of these events correspond to the effective bound and unbound state lifetimes of the particles[7,8].

Figure S7 investigates how state lifetimes change over time in a competitive GA t-BPM sensor without analyte in solution and without fluid exchange. Figures S7A and B show how the distribution of the bound and unbound state lifetimes varies with the aging time, while Figure S7C summarizes the observed lifetimes. The data reveal that the bound-state lifetime remains unaffected by aging, suggesting that the nature of the bound state remains stable. However, the unbound state lifetime shifts towards longer lifetimes as a function of the aging time. This is in agreement with the interpretation that the slow signal changes in the t-BPM sensor result from the gradual dissociation of binders, predominantly from the sensing surface, as suggested by the single-sided aging experiments.

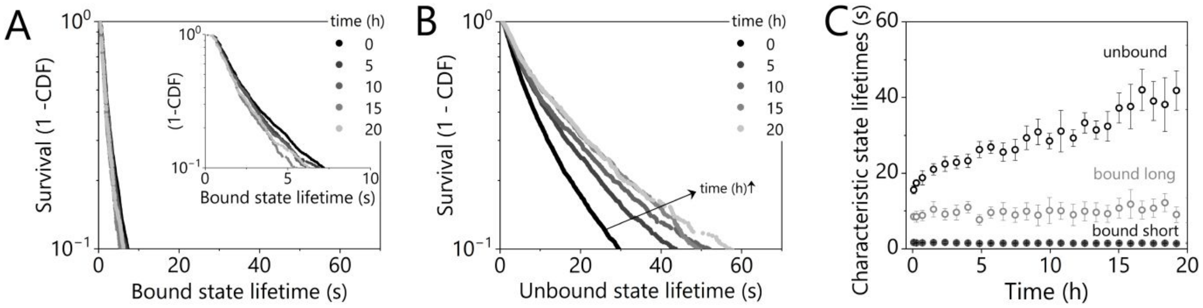

Figure S7. Analysis of state lifetimes in a competitive glycoalkaloid t-BPM sensor without analyte in solution and without fluid exchange. (A) Bound-state lifetime survival curves (1-CDF, with CDF being the cumulative distribution function) were measured over approximately 20 hours of continuous monitoring as a function of the aging time. The bound-state lifetime curves were fitted with a double-exponential decay function, allowing differentiation between specific interactions and a background contribution arising from nonspecific interactions or algorithm artifacts. The long characteristic lifetime, approximately 10 s, is attributed to the dissociation of single antibody–analogue bonds. (B) Unbound-state lifetimes were measured as a function of the aging time. These lifetimes were fitted with a multiexponential fit due to the heterogeneous distribution of binders on both particles and the sensing surface. The unbound state lifetimes correspond to the association process between particles and the sensing surface, which becomes progressively less effective over time. (C) Characteristic bound- and unbound-state lifetimes plotted as a function of time during approximately 20 hours of continuous sensor signal monitoring. The bound-state lifetimes (with short lifetimes shown as dark gray dots and long lifetimes shown as light gray open dots) remained constant, while the characteristic unbound-state lifetime (black open dots) increased over time.

S9. Sequential buffer flushing to remove leftover analogue molecules

Figure 1C in the main text depicts the signal of the t-BPM sensor under static conditions, in the absence of analyte in solution and without fluid exchange. The competitive GA t-BPM sensor exhibited a double exponential signal decrease, related to fast and slow changes. This behavior could be caused by several mechanisms, as listed in Figure 1D.

Another potential cause for a decrease of signal as a function of time could be the incomplete removal of unbound analogue molecules after the sensor activation step. The leftover analogue molecules could remain in solution and hybridize with the sensing surface over time, increasing the likelihood of particles binding multivalently to the sensing surface.

To investigate this hypothesis, Figure S8A presents a comparative experiment with different wash steps. Two GA t-BPM sensors were prepared, flushed with different volumes of buffer solutions at different time points. In experiment 1, after analogue solution incubation (5-10 minutes), the sensor was flushed once with 0.5 M NaCl in PBS (red arrow), and the signal was monitored under static conditions for approximately 10 hours. In experiment 2, after analogue removal using the same buffer solution (black arrow), the sensor underwent sequential buffer flushing (orange arrows) to eliminate any residual unbound analogue molecules that could potentially cause fast signal changes. Particle motion was not recorded during flushing but resumed afterward under static conditions.

Figure S7B compares the signals from the two experiments. Both exhibited a double-exponential signal loss, consistent with the behavior observed in Figure 1C. The signal loss in the sequentially flushed sensor (light blue dots) was more pronounced than in the static sensor (dark blue dots), which underwent only a single washing step after analogue addition. These findings suggest that fast signal changes in the competitive GA sensor are not attributable to unbound analogue molecules hybridizing to the sensing surface over time. The origin of the stronger signal loss in the experiment with sequential flushing is not yet clear.

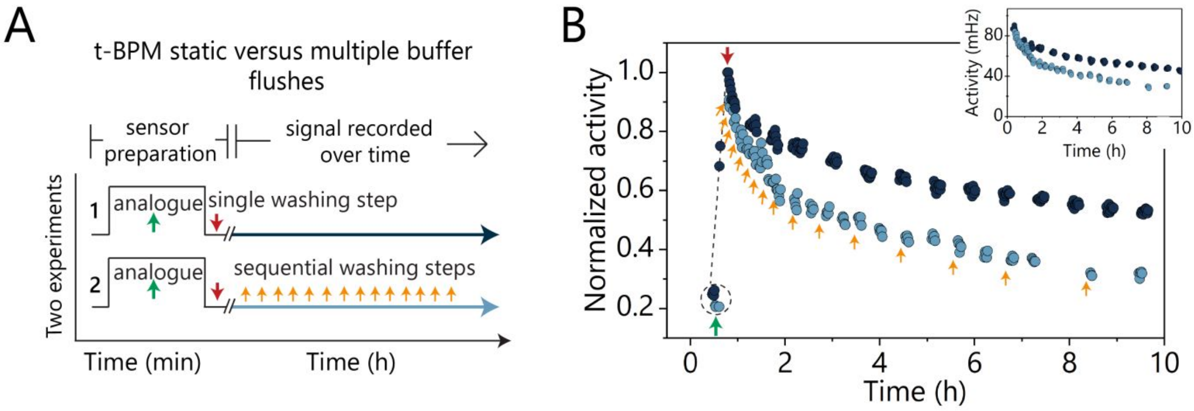

Figure S8. Presents the t-BPM sensor signal recorded under static conditions compared to the sensor signal following sequential buffer flushing intended to eliminate unbound analogue molecules from the fluidic chamber, which can potentially induce multivalent particle binding to the sensing surface. (A) Experimental protocols were designed to assess the effect of buffer flushing on the removal of unbound analogue molecules from the fluidic chamber and its effect on signal stability over time. Two distinct experiments were performed in separate flow cells. In Experiment 1, the fluidic chamber was incubated with an analogue solution, followed by a single buffer wash (indicated by a red arrow) to remove unhybridized analogue molecules. The sensor signal was subsequently recorded under static conditions without further buffer solution exchange. In Experiment 2, the fluidic chamber underwent a similar incubation with analogue solution, but sequential buffer flushing (orange arrows) was performed to thoroughly remove residual analogue molecules. During the flushing process, particle motion was not recorded, but particle motion was recorded after completing the flushing process. (B) In both experiments, sensor signals were recorded over approximately 10 hours following sensor preparation steps as described in panel A. Data points represents a one-minute measurement interval. Activity values were normalized based on the sensor's baseline, as detailed in the caption of Figure 4. Buffer flushing with buffer was performed at a flow rate of 100 μL/min for 2 min.

S10. Effectiveness of oligo blocker

Control experiments (Figure S8) involving sequential buffer flushing suggest that free analogue molecules remaining in solution are not the primary cause of fast signal changes. Instead, it is hypothesized that analogue molecules quickly bind to the sensing surface near the particle, creating a high local density that facilitates multivalent particle binding.

To prevent this effect and slow down fast signal changes, a non-functionalized oligonucleotide blocker with the same sequence as the analogue molecule was employed. The oligo blocker aims to partially block the sensing surface, reducing localized high densities of analogue molecules. Figure S9 investigates its effectiveness. Figure S9A outlines three experiments designed to investigate the role of oligo blocker in reducing fast signal changes. In experiment 1, the fluidic chamber was flushed with analogue solution, followed by a washing step. In experiment 2, the sensing surface was first activated with analogue solution, followed by partial blocking with the oligo blocker. Unbound molecules were washed away before tracking the particle motion. In experiment 3, the sensing surface was first flushed with oligo blocker solution before flushing the analogue solution, followed by a buffer wash to remove unbound molecules.

Figure S9B compares the sensor signals across these different experimental conditions outlined in Figure S9A. Experiments 1 and 2 exhibited a similar signal loss as in Figure 1C, indicating that in Experiment 2, the oligo blocker did not effectively bind to the sensing surface after analogue addition and failed to slow down fast signal changes. In contrast, Experiment 3 exhibited no signal increase after analogue addition, suggesting that the oligo blocker successfully blocked the sensing surface, preventing analogue hybridization and sensor activation.

These findings indicate that analogue molecules rapidly hybridize with capture molecules on the sensing surface, likely forming localized dense regions that render subsequent oligo blocker addition ineffective. Conversely, the absence of signal increase in Experiment 3 suggest that the oligo blocker effectively hybridized with capture molecules, preventing the hybridization of analogue molecules. In summary, for effective t-BPM sensor activation and partial blocking, analogue and oligo blocker molecules must be simultaneously added. Sequential addition either fails to block the surface or inhibits sensor activation due to the quick hybridization of the oligonucleotides.

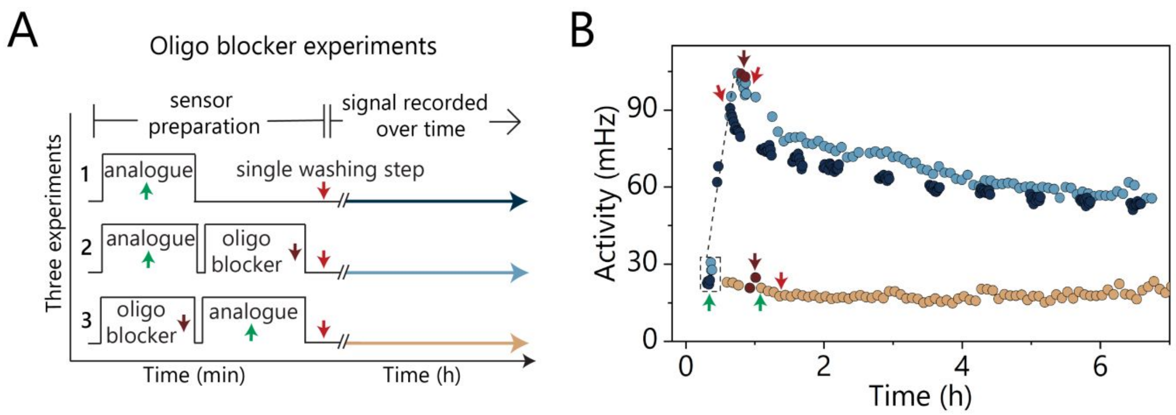

Figure S9. Control experiments evaluate the role of an oligo blocker as a sensing surface-blocking molecule to minimize fast signal changes in the competitive GA sensor. The experiments were conducted under static conditions, without analyte in solution and without fluid exchange. (A) Three distinct protocols were investigated to understand the effect of the sequence of addition of the analogue and the oligo blocker during the preparation of the sensor. Each experiment was performed in separate flow cells to examine the influence of the oligo blocker and the order of molecular addition (analogue and blocker oligo). After sensor preparation, the particle switching activity was monitored over 20 hours. In Experiment 1: The fluidic chamber was incubated with the analogue solution, followed by a buffer wash to eliminate non-hybridized analogue molecules. In Experiment 2: An analogue solution was incubated in the fluidic chamber, followed by the addition of a blocker oligo solution. A subsequent washing step then removed the non-hybridized analogue and oligo blocker molecules. In Experiment 3: The fluidic chamber was initially incubated with the blocker oligo solution, and subsequently flushed with the analogue solution. A final buffer wash removed any unbound oligo blocker and analogue molecules from the chamber. (B) Sensor signals were recorded over approximately 20 hours following the experimental preparations outlined in panel A. Each data point represents a measurement period of 1 minute.

S11. Duplicate measurements of partial blocking strategies

Figure 4 in the main text elucidates the effects of partial blocking on both particles and the sensing surface, employed to explore the mechanisms underlying fast signal changes in the competitive t-BPM sensor.

Here, we report the fitting parameters from the measurements in Figure 4, along with duplicate measurements conducted for each of the four experimental outlined in Figure 4B.

Table S1 summarizes the fitting parameters for raw and normalized data for the four distinct experiments fitted with a double-exponential function,

$$y = A_0 + A_1 \cdot \exp(-k_1 \cdot x) + A_2 \cdot \exp(-k_2 \cdot x)$$

Table S1. Fit parameters of the different experiments with blocking strategies

Blocking strategies	A_0 *(mHz)*	A_1 (mHz)	A_2 (mHz)	k_1 (h^{-1})	k_2 (h^{-1})	r^2
			Raw data			
analogue	15.3	9.1	39.3	1.28 ± 0.12	0.070 ± 0.004	0.98
analogue/oligo blocker	22.0	4.3	21.6	0.68 ± 0.15	0.069 ± 0.009	0.97
analogue/analyte	15.9	14.4	45.7	0.76± 0.11	0.067 ± 0.018	0.99
analogue/oligo blocker/analyte	30.2	16.9	39.0	0.62 ± 0.05	0.049 ± 0.013	0.97
			Normalized data			
analogue	0.24	0.14	0.62	1.28 ± 0.12	0.070 ± 0.004	0.98
analogue/oligo blocker	0.46	0.09	0.45	0.68 ± 0.15	0.069 ± 0.009	0.97
analogue/analyte	0.21	0.19	0.60	0.76 ± 0.11	0.067 ± 0.018	0.99
analogue/oligo blocker/analyte	0.36	0.19	0.45	0.62 ± 0.06	0.049 ± 0.013	0.97

The decay rates indicate that blocking strategies effectively slow down fast signal changes in the t-BPM sensor, as reflected by the lower k_1 values observed in experiments 2, 3, and 4, compared to experiment 1, which lacked blocking of particles and the sensing surface. However, the blocking strategies did not significantly influence the slow signal changes, as reflected by the similar magnitudes of the k_2 values across all four experiments.

Figure S10 shows the normalized duplicate measurements for each blocking condition, as described in Figure 4B. Figure S10A presents the results for the t-BPM sensor without any blocking step. Figure S10B illustrates the duplicate measurement for partial blocking on the sensing surface using an oligo blocker, whereas Figure S10C depicts the outcomes for temporary antibody blocking on the particles. Figure S10D shows the duplicate measurements of the t-BPM sensor wherein both partial blocking approaches were concurrently implemented.

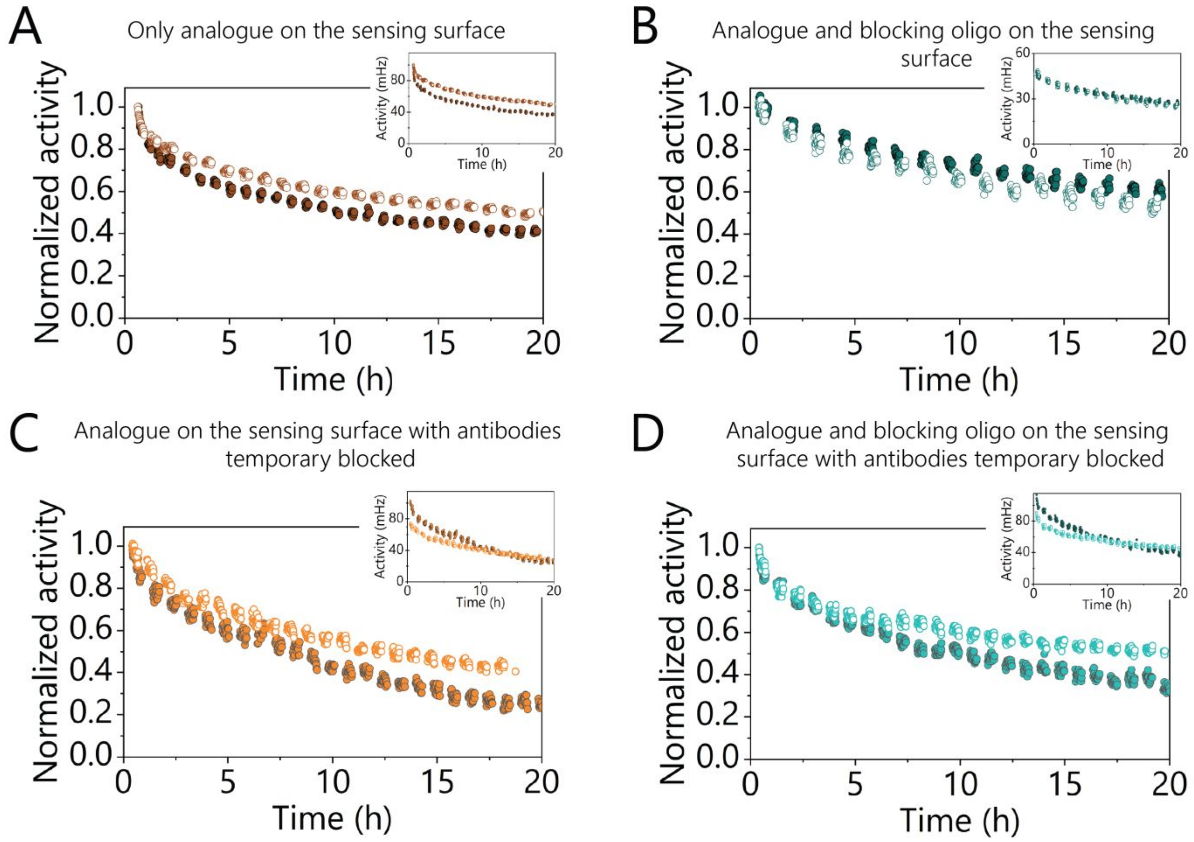

Figure S10. Duplicate measurements from four distinct experiments designed to investigate the underlying mechanisms of fast signal changes in the competitive GA sensor. Each experiment started with specific preparation steps, followed by a 20-hour measurement of switching activity. (A) The analogue solution was flushed to the fluidic chamber, followed by a washing step with buffer to remove unhybridized analogue molecules. (B) A mixture of analogue and oligo blocker solution was flushed into the chamber, followed by a washing step to remove the unhybridized analogue and oligo blocker molecules. (C) The analogue solution was flushed into the fluidic chamber, followed by the addition of a high-concentration analyte solution, and then a washing step to remove the analyte molecules. (D) A mixture of analogue and oligo blocker solution was flushed into the chamber, followed by the addition of a high-concentration analyte solution, and subsequent washing step to remove the analyte molecules. Sensor signals were measured over time following the preparation detailed in Figure 4B and the resulting curves were fitted with a double exponential fit.

S12. Distribution of particles for sensor with blocking strategies

Figure 3 in the main text presents the analysis of particle motion pattern and switching activity in a competitive glycoalkaloid (GA) t-BPM sensor under static conditions, with no analyte in solution or fluid exchange.

Figure S11 examines the same properties for sensors prepared with different blocking strategies, as detailed in Figure 4B. Figure S11A shows the relative distribution of particles in group 3 across four experiments, each employing different blocking strategies. Results reveal that the particle population growth in group 3 strongly depends on the applied blocking strategy. The sensor without any blocking strategy exhibited the largest increase in group 3, while sensors prepared with either partial surface blocking or temporary particle blocking showed more moderate increases. Among these sensors, sensors with partial surface blocking (experiments 2 and 4) demonstrated the lowest increase in group 3, whereas temporary blocking of particles resulted in a higher increase, though still lower compared to the sensor without any blocking strategy. The right panel of Figure S11A shows a sharp increase in group 3 particle population for the sensor without any blocking, within the initial hours of particle tracking, attributed to particles becoming multivalently bound to the sensing surface. In contrast, sensors with blocking strategies exhibited lower rates of increases in group 3.

Figure S11B depicts the relative distribution of non-switching particles in group 3, which represents particles transitioning from a switching state to a confined state due to the multivalent binding of particles to the sensing surface. All four experiments showed an increase in group 3 particle population over time, though the degree of increase varied depending on the blocking strategy. The sensor without any blocking strategy and the sensor with a temporary blocking of antibodies on particles exhibited the largest increases in this population. Conversely, sensors with partial surface blocking showed a smaller increase, suggesting that the blocking of the sensing surface reduces the likelihood of multivalent particle binding.

Figure S11C displays the relative distribution of non-switching particles in group 1. Across all the sensors, this population showed a gradual increase over the measurement period. This increase is attributed to the continuous loss of specific binders, primarily from the sensing surface.

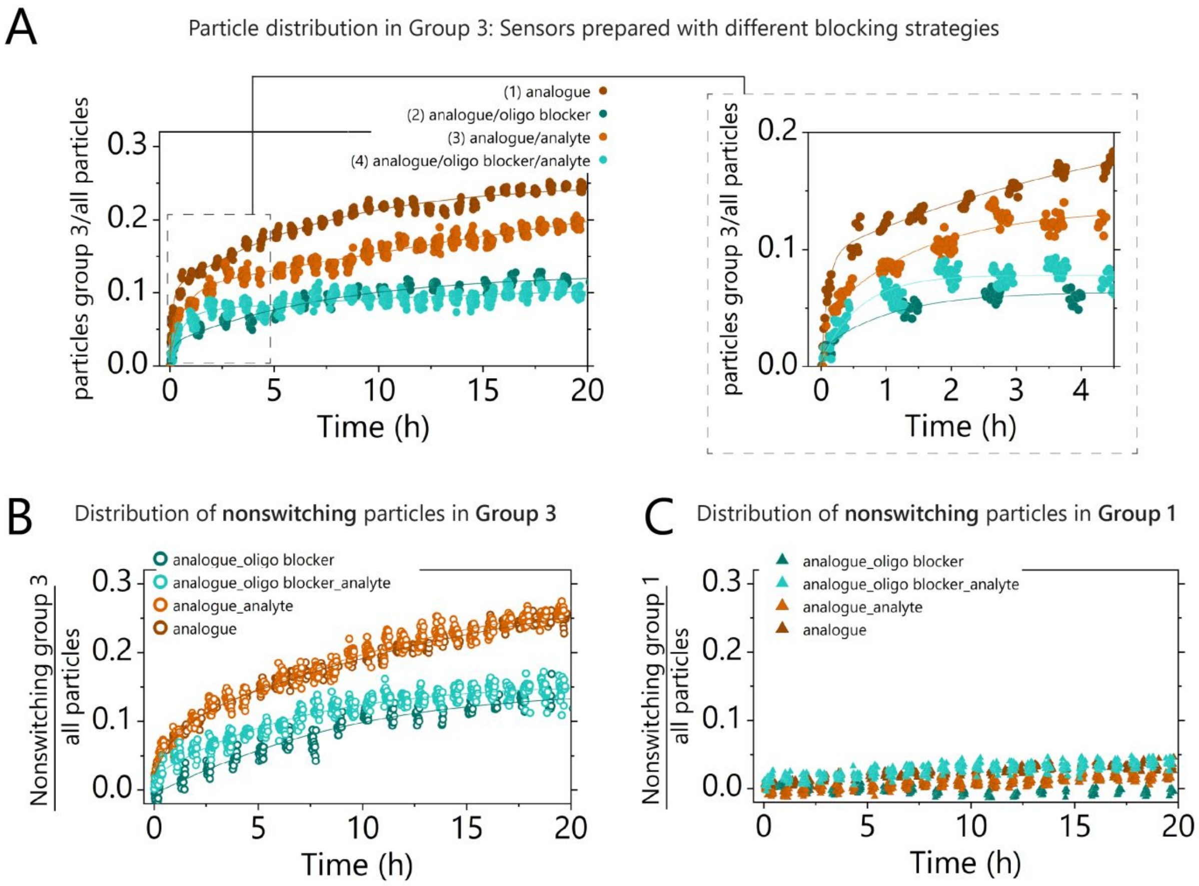

Figure S11. Distributions of particle populations based on their motion patterns and switching behavior analyzed over 20 hours for GA t-BPM sensors using different blocking strategies, measured without analyte in solution and without fluid exchange. (A) Relative number of particle in group 3 for the four sensors prepared with different blocking strategies, as detailed in Figure 4B. The right panel highlights particle population increases within the initial hours of particle tracking. (B) Relative number of particles exhibiting no switching events within group 3. (C) The relative number of particles showing no switching events within group 1.

References

(1) Cajigas, S.; De Jong, A. M.; Yan, J.; Prins, M. W. J. Molecular Origins of Long-Term Changes in a Competitive Continuous Biosensor with Single-Molecule Resolution. *ACS Sens* **2024**, *9* (7), 3520–3530. https://doi.org/10.1021/acssensors.4c00107.

(2) Visser, E. W. A.; Van Ijzendoorn, L. J.; Prins, M. W. J. Particle Motion Analysis Reveals Nanoscale Bond Characteristics and Enhances Dynamic Range for Biosensing. *ACS Nano* **2016**, *10* (3), 3093–3101. https://doi.org/10.1021/acsnano.5b07021.

(3) Visser, E. W. A.; Yan, J.; Van IJzendoorn, L. J.; Prins, M. W. J. Continuous Biomarker Monitoring by Particle Mobility Sensing with Single Molecule Resolution. *Nat Commun* **2018**, *9* (1), 2541. https://doi.org/10.1038/s41467-018-04802-8.

(4) Lin, Y. T.; Vermaas, R.; Yan, J.; De Jong, A. M.; Prins, M. W. J. Click-Coupling to Electrostatically Grafted Polymers Greatly Improves the Stability of a Continuous Monitoring Sensor with Single-Molecule Resolution. *ACS Sens* **2021**, *6* (5), 1980–1986. https://doi.org/10.1021/acssensors.1c00564.

(5) Bergkamp, M. H.; Cajigas, S.; van IJzendoorn, L. J.; Prins, M. W. J. High-Throughput Single-Molecule Sensors: How Can the Signals Be Analyzed in Real Time for Achieving Real-Time Continuous Biosensing? *ACS Sens* **2023**, *8* (6), 2271–2281. https://doi.org/10.1021/acssensors.3c00245.

(6) Yan, J.; Van Smeden, L.; Merkx, M.; Zijlstra, P.; Prins, M. W. J. Continuous Small-Molecule Monitoring with a Digital Single-Particle Switch. *ACS Sens* **2020**, *5* (4), 1168–1176. https://doi.org/10.1021/acssensors.0c00220.

(7) Lubken, R. M.; De Jong, A. M.; Prins, M. W. J. Multiplexed Continuous Biosensing by Single-Molecule Encoded Nanoswitches. *Nano Lett* **2020**, *20* (4), 2296–2302. https://doi.org/10.1021/acs.nanolett.9b04561.

(8) Van Smeden, L.; Saris, A.; Sergelen, K.; De Jong, A. M.; Yan, J.; Prins, M. W. J. Reversible Immunosensor for the Continuous Monitoring of Cortisol in Blood Plasma Sampled with Microdialysis. *ACS Sens* **2022**, *7* (10), 3041–3048. https://doi.org/10.1021/acssensors.2c01358.
